# Success of femtosecond laser-assisted deep anterior lamellar keratoplasty (Femto-DALK): a clinical case series of 42 eyes using an intraoperative optical coherence tomography (iOCT)-guided laser-generated pre-descemet tunnel

**DOI:** 10.1186/s12886-026-05416-1

**Published:** 2026-10-14

**Authors:** Nicola R. Otto, Friedrich A. Steindor, Gerd Geerling, Theo G. Seiler

**Affiliations:** 1 Department of Ophthalmology, Medical Faculty and University Hospital Düsseldorf, Heinrich-Heine University Düsseldorf, Moorenstr. 5, 40225 Düsseldorf, Germany; 2 Institut für Refraktive and Ophthalmo-Chirurgie (IROC), Stockerstr. 37, Zürich, 8002 Switzerland; 3 Department of Ophthalmology, Inselspital, Bern University Hospital, Bern, 3010 Switzerland

**Keywords:** Deep anterior lamellar keratoplasty, DALK, Femto-DALK, Big-Bubble, Femtosecond laser, Intraoperative OCT

## Abstract

**Purpose:**

To evaluate the success of Femto-DALK with an iOCT-guided generated intrastromal pre-Descemet tunnel.

**Methods:**

This monocentric, retrospective study evaluated all patients treated with Femto-DALK between January 2020 and December 2023 at the Department of Ophthalmology at the University of Düsseldorf (Germany). The iOCT-guided tunnel was generated 30 microns anterior of Descemet’s membrane. BB generation, surgery time and conversion to penetrating keratoplasty (PK) were analyzed.

**Results:**

A total of 42 eyes of 41 patients were identified. BB creation was successful in 78.6% of cases. Intraoperative conversion to PK was necessary in 28.6% of the eyes and microperforation of Descemet’s membrane (DM) occurred in 16.7% of completed DALKs. Mean operation time was 118.6 ± 40.68 min. Among successful Femto-DALKs (*n* = 30), double anterior chamber was observed in 26.7%, and re-bubbling was needed in 23.3%. Re-keratoplasty was performed in one eye (3.3%).

**Conclusion:**

Femto-DALK with an iOCT-guided pre-Descemet tunnel generation is a feasible technique that achieves BB success rates comparable to the upper range reported for manual dissection, without a clear demonstrated improvement over manual technique.

## Key messages

**What is known:**

- Deep anterior lamellar keratoplasty (DALK) is the preferred surgical approach for stromal corneal disease with a healthy endothelium but remains technically demanding.
- The Big-Bubble (BB) technique is the preferred method to bare Descemet’s membrane (DM), yet its success depends strongly on surgeon experience and on precise placement of the intrastromal pre-Descemet tunnel.

**What is new:**

- Femto-DALK with an iOCT-guided pre-Descemet tunnel achieves a BB-formation rate (78.6%) within the upper range reported for manual tunnel creation.
- iOCT-guided femtosecond laser (FSL)-assisted pre-Descemet tunnel creation appears to be a safe and feasible technique that may help to standardize BB-DALK. A superiority over manual tunnel creation remains to be demonstrated in comparative studies.

## Introduction

DALK is considered the preferred treatment approach for stromal corneal diseases with healthy endothelium, such as keratoconus, stromal scarring, and stromal dystrophies, sparing DM and endothelium [1, 2]. By preserving the patient’s own endothelium, DALK provides multiple advantages over PK including the elimination of endothelial rejection, longer graft survival, reduced allograft rejection, fewer postoperative epithelial problems and nonimmunological failure [2–9]. Additionally, as an extraocular procedure, DALK avoids the risks associated with open sky surgery [5] and significantly reduces the risk for secondary glaucoma development [9].

Despite its advantages, the number of DALK procedures performed in Germany has remained relatively low over the past decades [1, 10], most likely because the procedure is considered technically challenging with a relatively high rate of conversion to PK, long and unpredictable surgery time along with a steep learning curve [11].

The preferred method for DALK is the Big-Bubble technique described by Anwar [12]. This technique involves creating a smooth separation of the anterior stroma from the pre-Descemet-layer (PDL) or DM by injecting air through an intrastromal channel close to the DM [12]. The creation of the intrastromal tunnel for pneumatic dissection is a crucial and challenging step in DALK, as it needs to extend as close as possible to the PDL and thus carries the risk of DM perforation and the need for a conversion to PK [2, 13].

FSL has already been used in DALK to aid in lamellar and side cuts, improving wound healing and standardizing the technique by providing reproducible and precise cuts in customizable shapes [14–16]. Despite potential advantages, the creation of the intrastromal pre-Descemet tunnel for air injection is widely performed manually, most likely due to non-availability of a FSL generating a pre-Descemet tunnel. The success rate for achieving a BB with a manual approach depends highly on the surgeon’s experience as well as the underlying corneal pathology, and ranges widely from 36.4 to 87% [17–20], however, most likely around 60% according to the Moorfields multisurgeon study including 326 eyes treated with the BB technique [18]. FSL technology has been proposed to assist in creating the intrastromal pre-Descemet guiding tunnel, enabling pneumatic dissection in DALK [21]. Recently, advanced software, combined with integrated iOCT allows for accurate intrastromal tunnel placement near DM [19, 21–25]. Pasricha et al. demonstrated that BB formation success is dependent on the distance between the air injection and DM [26]. This emphasizes the importance of a well-positioned pre-Descemet tunnel, which is facilitated by the presented approach.

Although previous studies have demonstrated promising results, including high rates of BB formation and minimal intraoperative complications and conversions to PK, their findings are often based on porcine eyes, ex vivo donor corneas, or small case series [19, 21–25]. This study provides real-life data from a single center performing Femto-DALK with an OCT-guided pre-Descemet intrastromal tunnel.

## Methods

In this retrospective study, we included all patients treated with Femto-DALK using the Ziemer LDV Z8 FSL (Ziemer Ophthalmic Systems, Port, Switzerland) between January 2020 and December 2023 at the Department of Ophthalmology at the University of Düsseldorf, Germany. All surgeries were conducted by the same surgeon (TGS).

The study was approved by the Ethics Committee of the Faculty of Medicine at Heinrich Heine University in Düsseldorf on February 21, 2024 (study number 2024–2772) and was conducted in accordance with the principles of the Declaration of Helsinki.

From the department’s digital database, we extracted medical history, surgery indication, intraoperative parameters (BB-formation, perforation of DM and conversion rate, duration of surgery) and postoperative outcomes.

### Preparation of the donor graft

Donor corneas were provided by Lions Eye Bank North-Rhine-Westphalia. We used the Ziemer LDV Z8 and the company’s artificial anterior chamber to cut the donor cornea with a diameter of 8.2 mm in an applanating manner.

### Surgical procedure

**Fig. 1:**
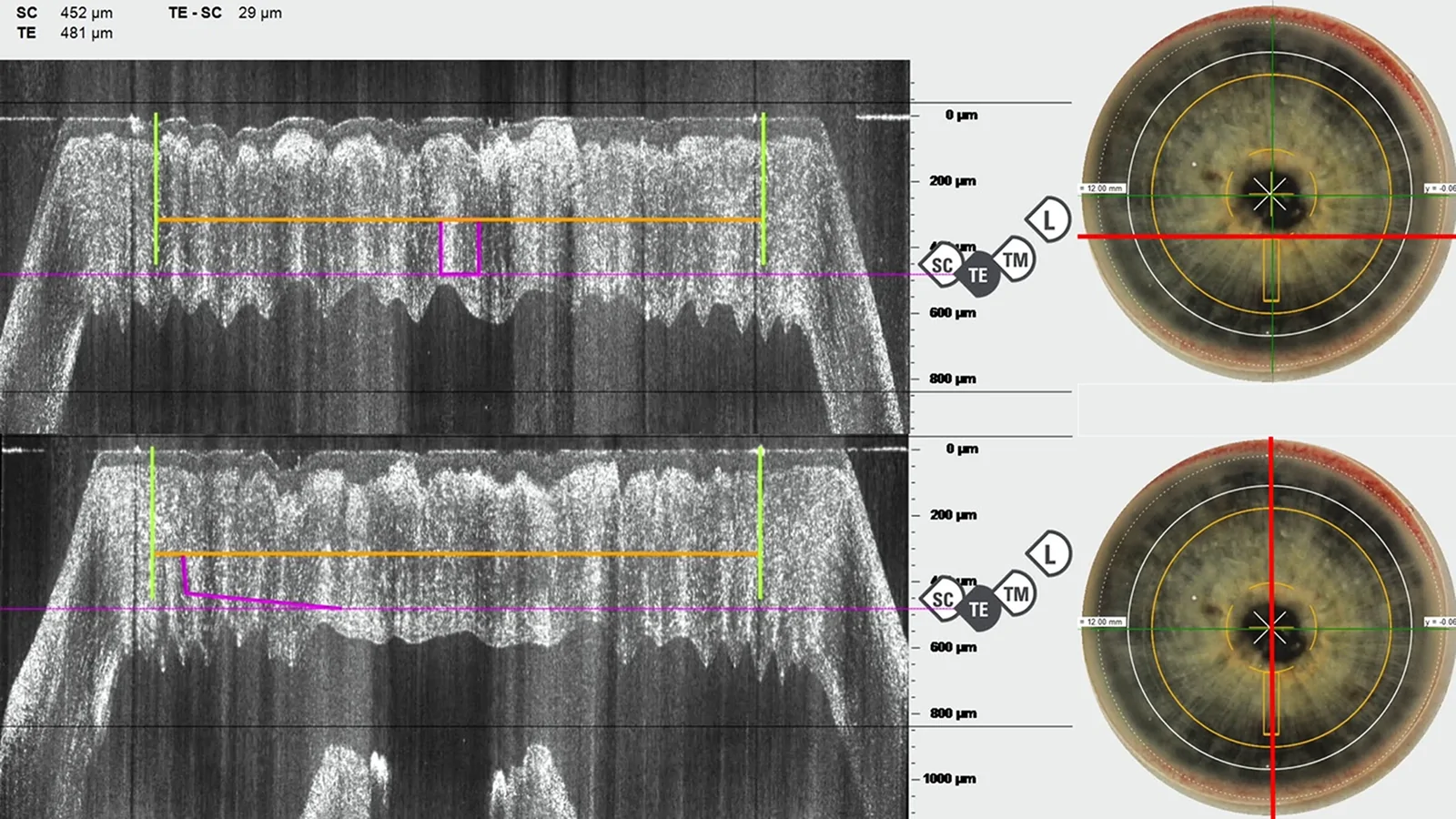
Intraoperative OCT imaging, with the B-Scan on the left-hand side and a live image of the eye on the right-hand side. The upper part of the image displays the horizontal axis and the lower part displays the vertical axis with a red line, which corresponds to the B-scan next to it. The pre-Descemet tunnel cut, planned using OCT-guidance, is marked by violet lines, the side cuts by green lines, and the lamellar cut by a yellow line. The Ziemer Z8 platform (Ziemer Ophthalmic Systems, Port, Switzerland) allows for manual repositioning of the cuts

Before surgery, we assessed corneal diameter as well as minimal and mid-peripheral pachymetry and performed a Nd: YAG-iridotomy at the 6 o’clock position. All surgeries were conducted under general anesthesia. After disinfection with diluted iodine solution, an eyelid speculum was placed, the geometric center of the patient’s cornea was marked, and the laser was docked using an applanating interface with 500 mmHg vacuum suction. An 8.0 mm trephination was centered on the cornea. The lamellar dissection plane was set 200 microns anterior to the thinnest preoperative pachymetry measured by OCT and further adjusted in very thin corneas. Side-cut extension was set to 200 microns anterior to DM using the iOCT (Fig. 1, green line) and refined if required. A second OCT-scan guided placement of the pre-Descemet tunnel using the standard laser settings (position 90°, angle 60°, length 1.8 mm, width 0.6 mm, laser power 180%, velocity 5.0 mm/s) (Fig. 1, red lines). Depending on the thinnest corneal location, the tunnel was shifted laterally and adjusted to run almost parallel to DM, ending approximately 30 μm anterior (Fig. 1, violet line).

**Fig. 2:**
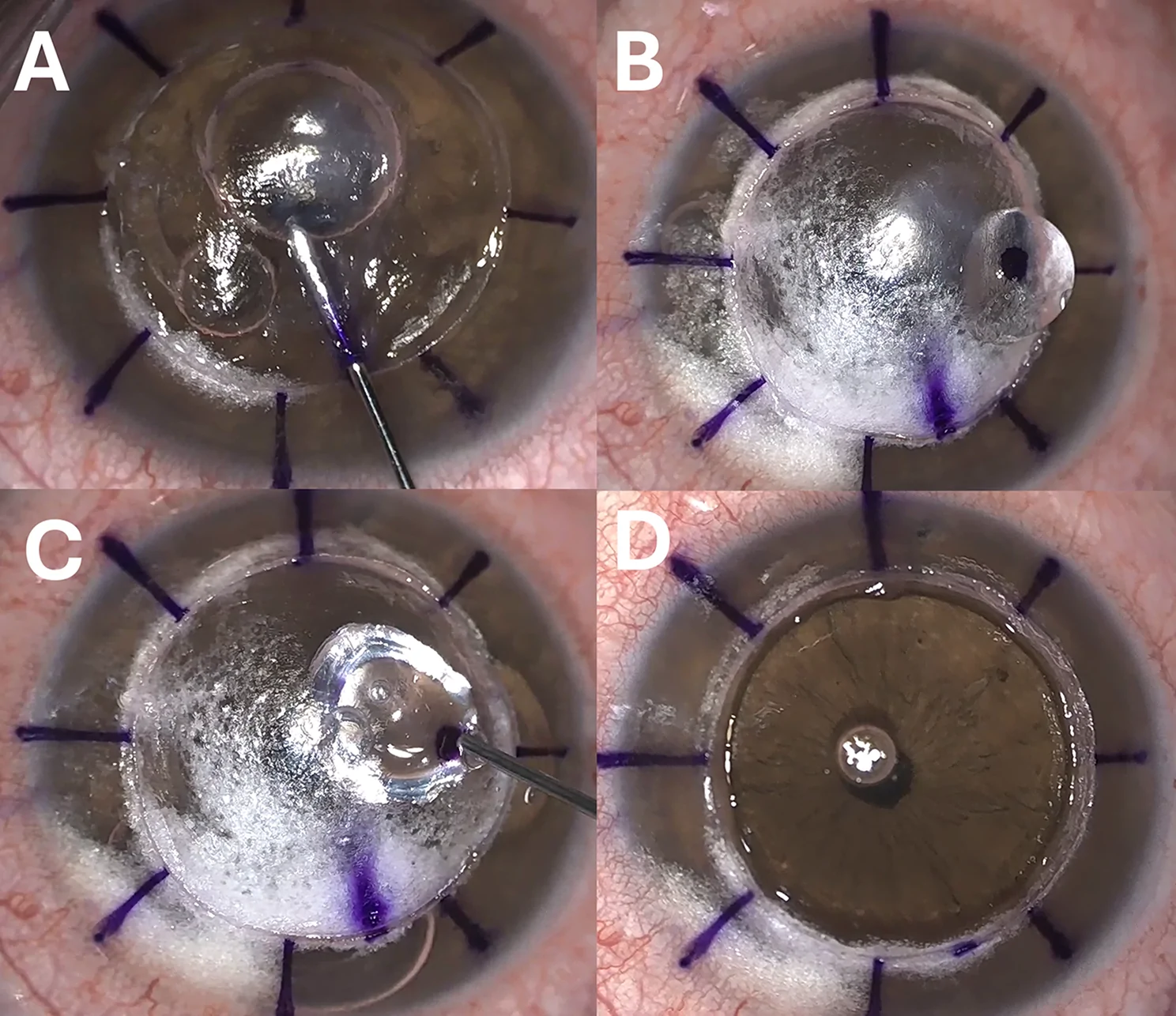
Intraoperative images of a successfully performed DALK procedure. A 27-gauge Fogla cannula is inserted into the pre-Descemet tunnel to create a successful Big-Bubble (**A**, **B**). The Big-Bubble is then incised and viscoelastic is injected into the lamellar space (**C**). After the overlying stroma is removed, the bare Descemet’s membrane is fully exposed (**D**)

After laser treatment, we performed a paracentesis, injected an air bubble into the anterior chamber and manually removed the tissue anterior to the laser dissection plane. After identification of the tunnel using a marked spatula, a 27-gauge Fogla-cannula connected to an air-filled 2-ml syringe was advanced into the tunnel (Fig. 2A). Sterile air was injected to induce BB formation. Up to five additional attempts were made if necessary. If BB formation failed, stromal dissection continued manually, using a spiral-down technique. After successful BB formation, the cornea was marked and viscoelastic was applied (Fig. 2B) to avoid air leakage. The BB was punctured with a 20G angled paracentesis knife and viscoelastic was injected into the lamellar space (Fig. 2C). The remaining tissue bridges were separated manually, the residual stromal layers were removed using keratoplasty scissors, and the interface was rinsed with balanced salt solution (Fig. 2D).

After donor DM removal, the graft was secured with eight interrupted cardinal sutures followed by two continuous running sutures (both 10 − 0 nylon, Alcon, Fort Worth, USA). The interrupted sutures were removed, the running suture knots buried, and suture tension was adjusted with a Placido disc to minimize astigmatism. Finally, either air or 20% SF6 was injected into the anterior chamber.

At the end of surgery, all patients received subconjunctival dexamethasone (4 mg) and betamethasone sodium phosphate (4 mg). Postoperatively, topical dexamethasone 1 mg/ml was administered five times daily and tapered by one drop per month to one drop daily. Patients additionally received topical ofloxacin 3 mg/ml four times daily for five days or until epithelial healing and 0.1% hyaluronic acid eye drops five times daily.

### Statistical analysis

Statistical analyses were performed using GraphPad Prism 10.2.1 (GraphPad Software, Boston, MA, USA). Categorical variables were compared using Fisher’s exact test. Statistical significance was defined as *p* < 0.05.

## Results

Femto-DALK was performed in 42 eyes of 41 patients (66.7% were male, mean age was 40 ± 17 years). Indications included keratoconus (*n* = 29), stromal dystrophies (*n* = 6), stromal scars (*n* = 6), and post-laser-assisted in situ keratomileusis (LASIK) keratectasia (*n* = 1). Previous ophthalmologic interventions had been performed in eleven patients, including corneal crosslinking (*n* = 7), surface excimer-ablation (Phototherapeutic/Photorefraktive Keratectomy [PRK/PTK]; *n* = 4), and LASIK (*n* = 2), two having received multiple interventions. Comorbidities included atopic dermatitis (*n* = 3), previous herpes keratitis (*n* = 1), and trisomy 21 (*n* = 1) (Table 1).

Mean preoperative minimal corneal thickness was 362.4 ± 112.3 μm. Advanced stromal scarring was present in 27 eyes (64.3%), with a mean scar-depth/minimal corneal thickness ratio of 0.68 ± 0.24 (Table 1). Stromal scarring was not associated with BB formation or conversion to PK.

**Table 1:** Preoperative patient and corneal parameters

**Patient Parameters (n = 42)**	
Gender (male: female)	28:14 (66.7%:33.3%)
Age (years)	40.0 ± 17.3
**Indications (** ***n*** ** = 42)**	
Keratoconus	29 (69.0%)
Stromal dystrophies	6 (14.3%)
Stromal scars	6 (14.3%)
Post-LASIK keratectasia	1 (2.4%)
**Previous ophthalmologic Interventions (** ***n*** ** = 42)**	
Crosslinking	7 (16.7%)
Surface excimer-ablation	4 (9.5%)
LASIK	2 (4.8%)
**Other comorbidities (** ***n*** ** = 42)**	
Atopic dermatitis	3 (7.1%)
S/P Herpes keratitis	1 (2.4%)
Trisomy 21	1 (2.4%)
**Preoperative corneal parameters (** ***n*** ** = 37)**	
Minimal corneal thickness (µm)	362.4 ± 112.3 μm
**Patients with presence of corneal scarring (** ***n*** ** = 27)**	
Scar depth (µm anterior DM) (*n* = 26)	110.8 ± 92.9
Scar depth / minimal corneal thickness (*n* = 26)	0.68 ± 0.24

DM = Descemet’s membrane, µm = micrometers, LASIK = laser-assisted in situ keratomileusis. S/P = status post. SD = standard deviation. Data is given as mean (± SD) or absolute numbers with proportions

A BB was achieved in 33 eyes (78.6%). Overall, DALK was completed in 30 eyes (71.4%), whereas twelve procedures (28.6%) required intraoperative conversion to PK (Table 2).

Among successful DALKs, intraoperative micro-perforation of DM occurred in five eyes (16.7%). Postoperatively, eight eyes (26.7%) developed a double anterior chamber, requiring re-bubbling with 20% SF6 in seven cases. One graft (3.3%) failed within six months post-surgery and underwent PK. Mean cut-to-suture time for completed DALKs was 118.6 ± 40.68 min (Table 2).

**Table 2:** Intraoperative and postoperative parameters

**Intraoperative parameters (n = 42)**	
Big-Bubble achieved	33 (78.6%)
Conversion to PK	12 (28.6%)
**Intraoperative parameters for successful DALKs (** ***n*** ** = 30)**	
Perforation of DM	5 (16.7%)
Operation time (minutes)	118.6 ± 40.7
**Postoperative parameters for successful DALKs (** ***n*** ** = 30)**	
Double anterior chamber formation	8 (26.7%)
Re-bubbling	7 (23.3%)
Re-keratoplasty within 6 months	1 (3.3%)

PK = penetrating keratoplasty. DM = Descemet’s membrane. BB-DALK = Big-Bubble deep anterior lamellar keratoplasty, SD = standard deviation. Data is given as mean (± SD) or absolute numbers with proportions

## Discussion

Despite its advantages, DALK remains technically challenging and is still performed considerably less frequently than PK in Germany, with approximately 300 DALK procedures per year [10]. There is also no clear consensus among corneal transplant surgeons on the superiority of laser-assisted versus manual DALK [27]. One advantage of the FSL is the iOCT, which was pioneered by our group in 2005 and described as useful in other indications, and in particular the guided pre-Descemet tunnel to facilitate BB formation [21, 28–31].

Until recently, FSL technology in BB-DALK was limited to creating lamellar and side cuts, with the pre-Descemet tunnel still being prepared manually. More recently, however, several laser systems have incorporated the use of iOCT-guided pre-Descemet tunnel creation [19, 21–25].

Our BB rate falls within the upper range of the reported BB rate of 36.4–87% for manually created tunnels [17–20]. Likewise, the rate of intraoperative microperforation in successful DALKs in our study is consistent with the reported range of 6.4–39.5% for manual DALK [20, 32, 33]. Our conversion rate of 28.6% lies within the reported range of 0–60% [18, 20, 34]; however, it is relatively high, likely reflecting the surgeon’s learning curve and the high proportion of advanced stromal scarring. Advanced stromal scarring was present in 64.3% of eyes. Although stromal scarring has been associated with BB failure, DM perforation, conversion to PK, and postoperative development of double anterior chamber [21, 35–38], we found no association between the presence of stromal scarring and successful BB formation or conversion to PK despite a mean scar-depth/corneal-thickness ratio of 68%. Our rate of 26.7% of double anterior chamber formation lies at the upper end of the reported range of 1.2–30.2% after DALK [20, 33, 39, 40]. A potential source might be the injection of air instead of SF6 after surgery in eyes without micro-perforations.

Liu et al. were the first to experimentally evaluate the Ziemer LDV Z8 for a precise, controlled and reproducible one-step DALK technique, using 30 porcine and 3 human cadaver eyes to demonstrate the laser’s cutting accuracy with a target deviation of less than 10%, as well as the feasibility of pneumatic dissection. In a subsequent clinical case series of 14 eyes, successful BB was achieved in all cases, with no case of DM perforation or conversion to PK [21]. This is in accordance with the presented results, as no primary perforation due to the pre-Descemet tunnel was found.

Lucisano et al. and Singh et al. further explored the feasibility and initial outcomes of this novel technique, presenting a case series of eleven and ten eyes, respectively. In both studies, successful BB-formation was achieved in all cases, with no occurrences of DM perforation or conversion to PK [22, 24]. Additionally, no postoperative complications were reported [22].

Similarly, Abusayf et al. utilized the Ziemer LDV Z8 with integrated iOCT, focusing on a large-diameter mushroom-shaped cut pattern. Their clinical case series of ten eyes also demonstrated successful BB-formation in all cases, with no intraoperative complications or conversions. One eye in their study developed a transient double anterior chamber, which resolved spontaneously [25].

Pedrotti et al. further explored whether the sequence of performing first the tunnel dissection or the lamellar and side cuts influences BB formation. Using 20 human cadaver eyes, they performed the lamellar and side cuts first in ten eyes and the channel cut first in the other ten eyes. Their findings revealed a lower rate of BB formation and one case of DM perforation when the lamellar and side cuts were performed before the channel cut. This suggests that laser-induced gas generation may lead to inappropriate channel creation [23]. We followed their recommendation to cut the channel first in all our cases and can confirm that no primary perforation due to the generation of the pre-Descemet tunnel has occurred.

Malyugin et al. were the first to compare outcomes between laser-cut tunnel and manually dissected tunnels in a larger cohort. Their study included 28 eyes that underwent FSL-tunnel creation and 33 using a manual tunnel creation, demonstrating significantly higher rates of BB-formation (63.4% vs. 36.4%) and shorter operating times (21.8 ± 5.1 vs. 25.6 ± 6.8 min to bare DM). No significant differences were observed in the rate of DM perforation and conversion to PK (7.1% vs. 15.1%) [19]. Unlike our study, Malyugin et al. created a maximum channel depth of 90–120 μm anterior to DM [19], which may explain their lower rate of BB-formation (63.4% vs. our 78.6%).

## Conclusions

Our study adds to the growing body of evidence supporting the feasibility and safety of FSL-assisted iOCT-guided tunnel creation in BB-DALK. Although long operating time and high acquisition costs of the laser system must be critically balanced in terms of cost efficiency, the individual patient benefits from a successful operation regardless of costs and operating time.

In conclusion, FSL-assisted iOCT-guided tunnel creation in BB-DALK is a safe and feasible technique with outcomes comparable to manual dissection. Whether it offers a true improvement over manual technique remains to be established in larger, comparative studies.

## Data Availability

The datasets analyzed during the current study are not publicly available due to patient privacy/data protection regulations, but are available from the corresponding author on reasonable request.

## Abbreviations

BB: Big-Bubble
DALK: Deep anterior lamellar keratoplasty
DM: Descemet’s membrane
Femto-DALK: Femtosecond laser-assisted deep anterior lamellar keratoplasty
FSL: Femtosecond laser
iOCT: Intraoperative optical coherence tomography
OCT: Optical coherence tomography
LASIK: Laser-assisted in situ keratomileusis
PDL: Pre-Descemet-layer
PK: Penetrating keratoplasty
PRK: Photorefraktive Keratectomy
PTK: Phototherapeutic Keratectomy
SD: Standard deviation
